# Stable gastric pentadecapeptide BPC 157 can improve the healing course of spinal cord injury and lead to functional recovery in rats

**DOI:** 10.1186/s13018-019-1242-6

**Published:** 2019-07-02

**Authors:** Darko Perovic, Danijela Kolenc, Vide Bilic, Nenad Somun, Domagoj Drmic, Esmat Elabjer, Gojko Buljat, Sven Seiwerth, Predrag Sikiric

**Affiliations:** 1 Department of Pharmacology, School of Medicine, University of Zagreb, Salata 11, P.O. Box 916, 10000 Zagreb, Croatia; 2 Department of Pathology, School of Medicine, University of Zagreb, Salata 9, 10000 Zagreb, Croatia

**Keywords:** BPC 157, Injury, Rats, Spinal cord

## Abstract

**Background:**

We focused on the therapeutic effects of the stable gastric pentadecapeptide BPC 157 in spinal cord injury using a rat model. BPC 157, of which the LD1 has not been achieved, has been implemented as an anti-ulcer peptide in inflammatory bowel disease trials and recently in a multiple sclerosis trial. In animals, BPC 157 has an anti-inflammatory effect and therapeutic effects in functional recovery and the rescue of somatosensory neurons in the sciatic nerve after transection, upon brain injury after concussive trauma, and in severe encephalopathies. Additionally, BPC 157 affects various molecular pathways.

**Methods:**

Therefore, BPC 157 therapy was administered by a one-time intraperitoneal injection (BPC 157 (200 or 2 μg/kg) or 0.9% NaCl (5 ml/kg)) 10 min after injury. The injury procedure involved laminectomy (level L2-L3) and a 60-s compression (neurosurgical piston (60–66 g) of the exposed dural sac of the sacrocaudal spinal cord). Assessments were performed at 1, 4, 7, 15, 30, 90, 180, and 360 days after injury.

**Results:**

All of the injured rats that received BPC 157 exhibited consistent clinical improvement, increasingly better motor function of the tail, no autotomy, and resolved spasticity by day 15. BPC 157 application largely counteracted changes at the microscopic level, including the formation of vacuoles and the loss of axons in the white matter, the formation of edema and the loss of motoneurons in the gray matter, and a decreased number of large myelinated axons in the rat caudal nerve from day 7. EMG recordings showed a markedly lower motor unit potential in the tail muscle.

**Conclusion:**

Axonal and neuronal necrosis, demyelination, and cyst formation were counteracted. The functional rescue provided by BPC 157 after spinal cord injury implies that BPC 157 therapy can impact all stages of the secondary injury phase.

## Introduction

We focused on the application of the stable gastric pentadecapeptide BPC 157 [1–11] to improve the outcomes of spinal cord injury in rats.

Spinal cord injury generally involves the preclusion of neural relays across the lesion site and is thereby predictably associated with a lack of functional improvement [12, 13]. On the other hand, there is evidence that spinal cord injury triggers a cascade of secondary degenerative events that cause further damage to the injured area and induce local inflammation along with hemorrhage and edema [12, 13] and that the therapeutic agents imatinib (which has been shown to inhibit cytokine production and reduce hemorrhage, edema, and inflammation) [14] and ibuprofen initiate favorable axonal growth and functional recovery through Rho inhibition [15]. Likewise, there is favorable evidence to support the engraftment of neural stem cells [16] or bone marrow stromal cells [17] into the lesion site. However, there are disputes about the relevant applicability of this evidence [18, 19], particularly considering the low survival rate of bone marrow stromal cells transplanted into the contused adult rat spinal cord [20, 21] and the need to completely fill the lesion site with neural stem cells [22]. Consequently, there have been attempts to improve the therapeutic effectiveness with combined treatments (i.e., neural stem cells with fibrin and a growth factor cocktail (BDNF; NT-3; mGDNF; IGF; bFGF; EGF; PDF; aFGF; and HGF) [23] or bone marrow stromal cells with the application of cyclosporine, minocycline, and methylprednisolone [24]). Likewise, considering the beneficial effect of the deletion of the Nogo Receptor 1 (NgR1) gene, a sequential combination of Nogo-A suppression (by anti-Nogo-A antibody treatment) and treadmill rehabilitative training was examined [25].

It is generally believed that further attempts are fully justified [26]. In comparison, the stable gastric pentadecapeptide BPC 157, an emerging treatment with potential therapeutic applications, appears to be unrestricted by the limitations seen in previous therapies. The stable gastric pentadecapeptide BPC 157, an original cytoprotective antiulcer peptide that is used in ulcerative colitis and recently in a multiple sclerosis trial and that has an LD1 that has not been achieved [1–11], is known to have pleiotropic beneficial effects [1–11] and to interact with several molecular pathways [2, 27–32]. BPC 157 has beneficial effects on inflammation, hemorrhage, and edema after traumatic brain injury [33], various severe encephalopathies (which follow gastrointestinal and/or liver lesions), NSAID overdose [34–37], or insulin overdose seizures [38] and on severe muscle weakness after exposure to the specific neurotoxin cuprizone in a rat multiple sclerosis model [39] or magnesium overdose [40]. In other studies, it was shown that BPC 157 counteracts increased levels of proinflammatory and procachectic cytokines such as IL-6 and TNF-α [2]. Finally, BPC 157 improves sciatic nerve healing [41] when applied intraperitoneally, intragastrically, or locally at the site of anastomosis shortly after injury or directly into the tube after non-anastomosed nerve tubing (7-mm nerve segment resection).

Therefore, we used a model of spinal cord injury that has many characteristics found in human spastic syndrome [42] and can be used long-term to provide a realistic model of spasticity development in the tail muscle.

The administered therapy was a one-time intraperitoneal application of the stable gastric pentadecapeptide BPC 157, much like the one-time engraftment of neural stem cells [16] or bone marrow stromal cells [17] into the lesion site. This experiment will provide evidence that BPC 157 treatment can recover tail function, resolve spasticity, and improve neurologic recovery.

## Materials and methods

### Animals

Wistar albino male rats (aged 12 weeks, 350–400 g b.w.) were bred in-house (the animal facility at the Department of Pharmacology, School of Medicine, Zagreb, Croatia; registered by Directorate of Veterinary; Reg. No: HR-POK-007), acclimated for 5 days, and randomly assigned to experimental groups (at least 6 animals per experimental group and interval). The experiments were approved by the Local Ethics Committee. The laboratory animals were housed in PC cages in conventional laboratory conditions at a temperature of 22.4 °C, a relative humidity of 40–70%, and a noise level of 60 dB. Each cage was identified by the date, study number, group, dose, number, and sex of each animal. Fluorescent lighting provided illumination 12 h per day. A standard GLP diet and fresh water were provided ad libitum. Furthermore, all experiments were carried out under a blind protocol, and the effects were assessed by examiners who were completely unaware of the protocol. We certify that government regulations concerning the ethical use of animals were adhered to during this research.

### Drugs

The pentadecapeptide BPC 157 (GEPPPGKPADDAGLV, M.W. 1419) (Diagen, Ljubljana, Slovenia) dissolved in 0.9% NaCl was used in all experiments [1–11]. The peptide BPC 157 is part of the sequence of the human gastric juice protein BPC and is freely soluble in water and 0.9% NaCl at pH 7.0. BPC 157 was prepared as described previously with 99% high-pressure liquid chromatography (HPLC) purification, expressing 1-des-Gly peptide as an impurity [1–11].

### Surgery and spinal cord injury

Deeply anesthetized (3% isoflurane, ketamine 50 mg/kg b.w.) rats were subjected to laminectomy at lumbar level L2–L3, which corresponds to the sacrocaudal spinal cord (S2-Co1) as described previously [42]. A neurosurgical piston with a graduated force of 60–66 g was placed over the exposed dura and left for 60 s to induce a compression injury. After the piston was removed, the muscle and skin incisions were closed. A single injection (0.9% NaCl 5 ml/kg b.w.; pentadecapeptide BPC 157 200 μg/kg b.w. or 2 μg/kg b.w.) was administered intraperitoneally 10 min postinjury. Thereafter, the animals were returned to their cages in pairs, and food and water were provided ad libitum. According to previously assigned interval groups (7, 15, 30, 90, 180, and 360 days), the animals were sacrificed with an overdose of 3% isoflurane. To establish secondary spinal cord injury, four animals were sacrificed 10 min after spinal injury immediately prior to the administration of therapy. Four animals were subjected only to laminectomy without spinal cord injury and sacrificed after 360 days.

### Clinical evaluation

Tail motor function was scored 8 h and 1, 4, 7, 15, 30, 90, 180, and 360 days after injury (0—autotomy; 1—complete loss of tail function; 2—maximum elevation of 1/4 of the tail length; 3—maximum elevation of 1/2 of the tail length; 4—maximum elevation of 3/4 of the tail length; 5—normal function). At the same intervals, the tails were observed for spasticity; after manual stimulation with the standardized stretch/rub maneuver, the tails were scored according to the Bennett scale [42]: 0—normal phenotype; 1—flaccid tail; 2—hypertonic flexor muscle with coiled and stiff tail; 3—hyperreflexia, e.g., coiling flexor spasm and clonus in response to light touch or stretch; and 4—hypertonic flexor and extensor muscles, clonus and hyperreflexia, the latter including a positive curling reaction.

### Electrophysiology recordings

Before sacrifice, the animals from the 30-, 90-, 180-, and 360-day postspinal cord injury interval groups were placed in a wooden box with their tails exposed. Three pairs of monopolar needles were stabbed 3 mm deep into the tail 10, 60, and 100 mm caudal to the tail base. Using a TECA 15 electromyography apparatus with a signal filter between 50 Hz and 5 kHz, voluntary muscle activity was recorded from the most caudal pair of electrodes, and the average motor unit potential (MUP) was recorded. Thereafter, the compound motor action potential (CMAP) was recorded from the same pair of electrodes after stimulating the first and second electrodes (a repetition of 1 Hz and a stimulus duration of 0.05 ms). The amplitude, polyphasic changes, and the proximal and distal CMAP latencies were recorded, and the nerve conduction velocity was calculated according to previous studies [41, 43].

### Histology

A 10-mm long piece of the spinal column (the L2–L3 vertebral body) and the surrounding muscle were collected from each sacrificed animal and fixed in 4% formaldehyde in phosphate buffer (pH 7.4). Upon fixation, the spinal cord was decalcinated, dehydrated in graded ethanol solutions, and embedded in paraffin. Serial 5-μm cross-sections were deparaffinated in xylene, rehydrated in graded ethanol solutions, and stained with hematoxylin/eosin and toluidine blue (Kemica, Croatia). Part of the spinal cord gray and white matter was used for analysis under light microscopy (magnification × 300). According to previous studies [13, 33], the intensity and distribution of the following pathological spinal cord changes were evaluated semiquantitatively (0—no changes; 1—small or focal changes; 2—moderate changes; 3—numerous confluent changes): (a) the hemorrhagic zone, (b) edema, (c) the loss of neurons in anterior horn and intermediate gray matter, (d) vacuoles, and (e) the loss of lateral and posterior spinal column tracts.

For peripheral nerve analysis, a 5-mm-long piece of tail 15 mm distal from the tail base was collected from each sacrificed animal, fixed in 4% formaldehyde in phosphate buffer (pH 7.4), decalcinated, and impregnated with 1% osmium tetroxide for a few days. The specimens were dehydrated in graded ethanol solutions, embedded in paraffin, cut into 5-μm sections, deparaffinated in xylene, rehydrated in graded ethanol solutions, and mounted on glass slides. Representative field images of four caudal nerves from each tail were taken using light microscopy (magnification × 500) with a CCD camera using ISSA 3.1 software (VamStec, Zagreb) according to a previous study [41]. Axonal myelination was analyzed according to the following quantifications: (a) the total number of myelinated axons per 10,000 μm^2^; (b) the number of myelinated axons with a diameter ≥ 7 μm per 10,000 μm^2^; and (c) the average axonal diameter.

### Statistical analyses

Scoring data are expressed as the median, min, and max and were analyzed by Kruskal–Wallis ANOVA (*P* values < 0.05 were considered significant) followed by the Mann–Whitney *U* test (*P* values < 0.025 were considered significant) with Bonferroni correction; these tests are considered nonparametric alternatives to one-way ANOVA and Student’s *t* test. Numeric data are expressed as the mean ± standard deviation (SD) and were analyzed by one-way ANOVA followed by LSD test. The statistical program Statistica for Windows, ver. 12.1 (StatSoft Inc. Tulsa, OK, USA) was used for statistical analysis. *P* values < 0.05 were considered significant.

## Results

### Clinical examinations

#### Tail motor function score

As expected, the tail motor function scores demonstrated persistent debilitation in the rats that underwent spinal cord injury and received saline postinjury.

**Fig. 1:**
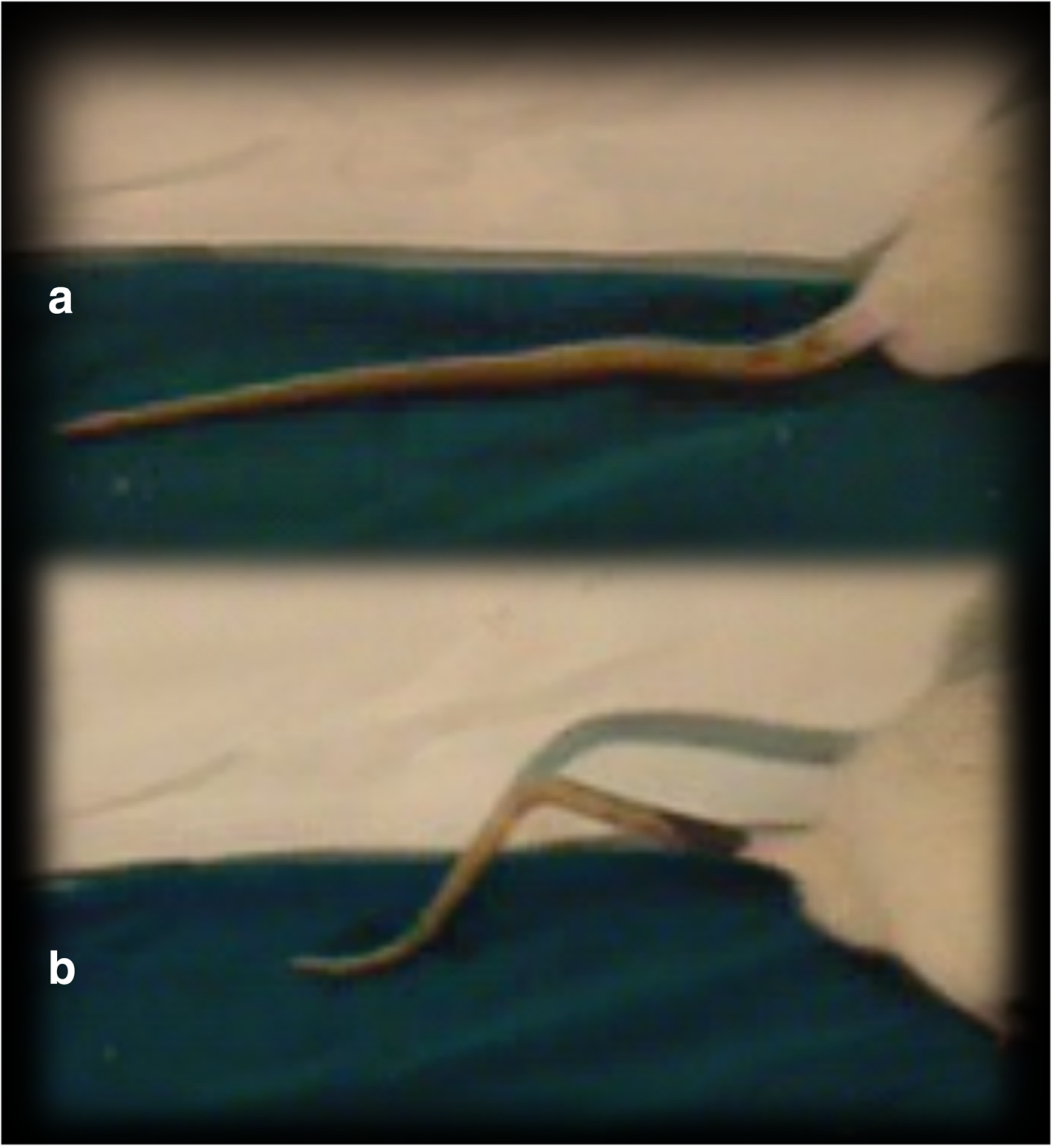
Tail motor function (**a**, **b**) in rats that underwent spinal cord injury. Thirty days following injury. Debilitated rats underwent spinal cord injury that received saline post-injury (**a**). Contrarily, rats that had received BPC 157 (**b**) exhibit tail motor function rescue and consistently better motor function than the corresponding controls (**a**)

**Fig. 2:**
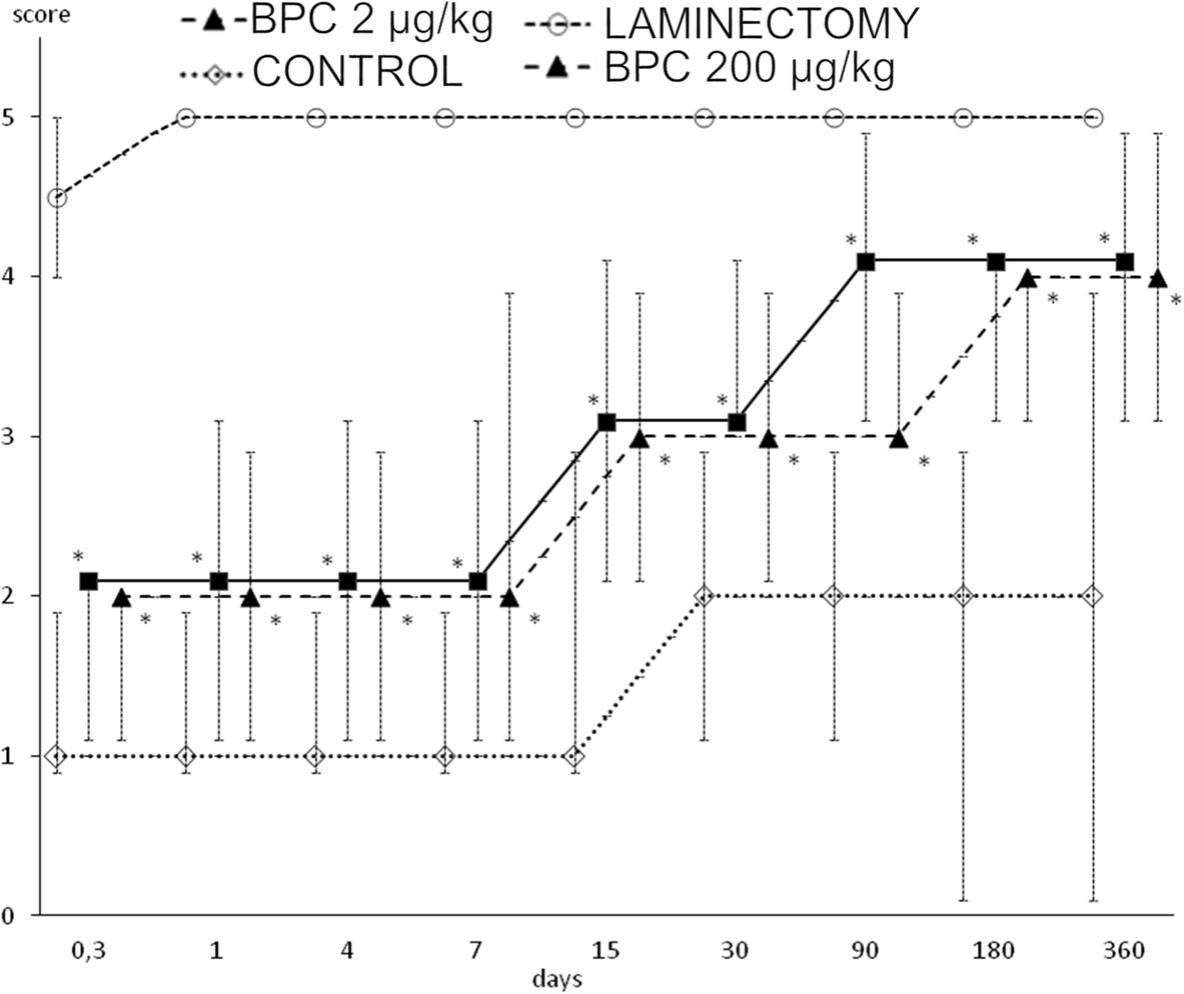
In rats that underwent spinal cord injury, debilitated and rescued tail motor function (BPC 157 (200 or 2 μg/kg) or saline 5 ml/kg intraperitoneally at 10 min after injury) presented by tail motor function score. Mark presents median score, and vertical bars correspond to maximum and minimum score. *P < 0.025 vs. control

In contrast, after initial disability, the rats that underwent spinal cord injury and received BPC 157 exhibited consistent improvement in motor function compared to that in the corresponding controls (Fig. 1). In particular, from day 180, autotomy was noted in the rats that underwent spinal cord injury but not in those that had been treated with BPC 157 (Fig. 2).

#### Tail spasticity

**Fig. 3:**
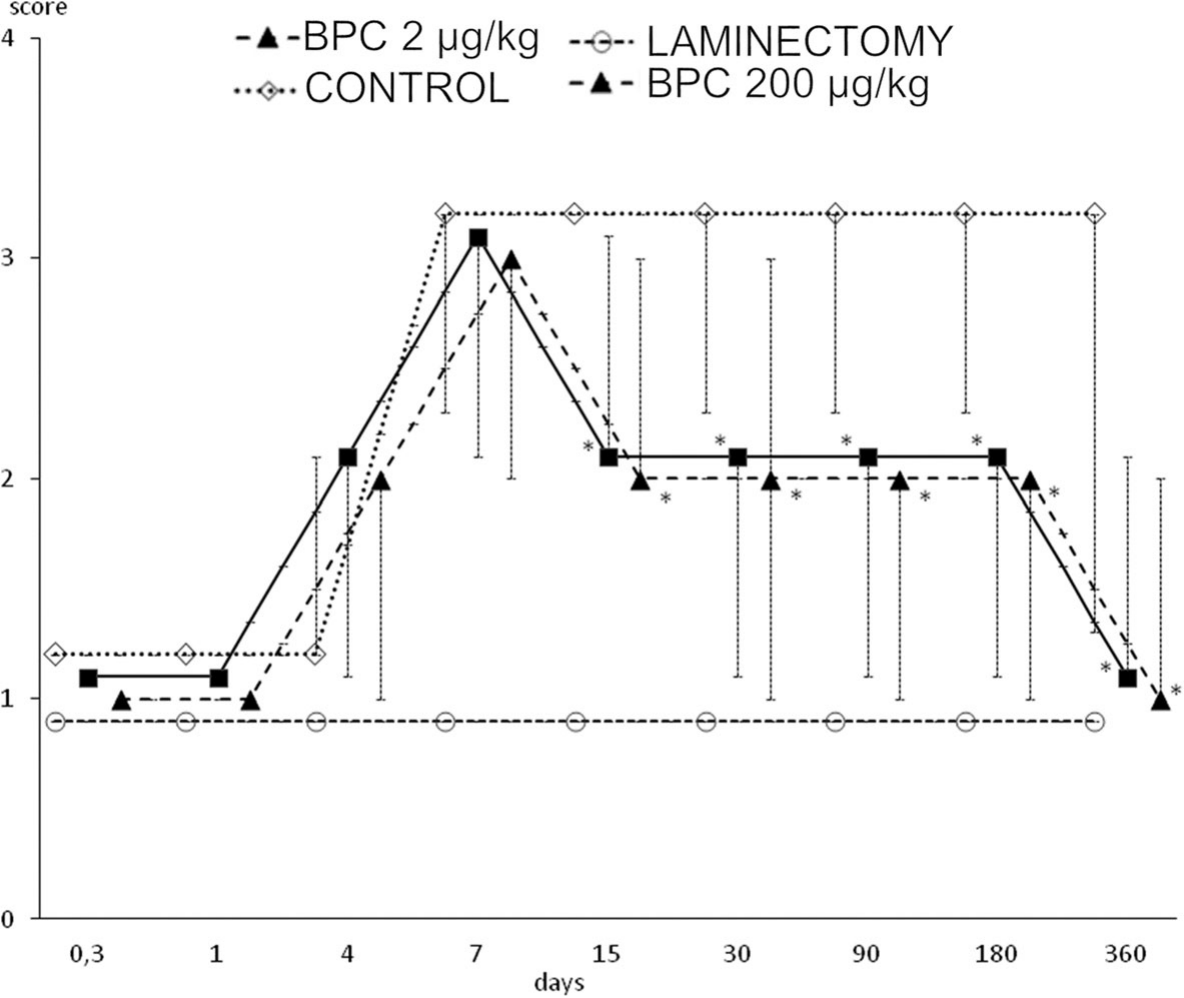
In rats that underwent spinal cord injury, debilitated and rescued tail spasticity (BPC 157 (200 or 2 μg/kg) or saline 5 ml/kg intraperitoneally at 10 min after injury) according to Bennett scoring. Mark presents median, and vertical bars correspond to maximum and minimum score. *P < 0.025 vs. control

Interestingly, the development of spasticity began earlier in the rats that underwent spinal cord injury and had been treated with BPC 157 than in the corresponding controls. However, the controls exhibited sustained spasticity until the end of the experiment (day 360) while the BPC 157 rats exhibited resolved spasticity by day 15 (Fig. 3).

### Histology results

**Fig. 4:**
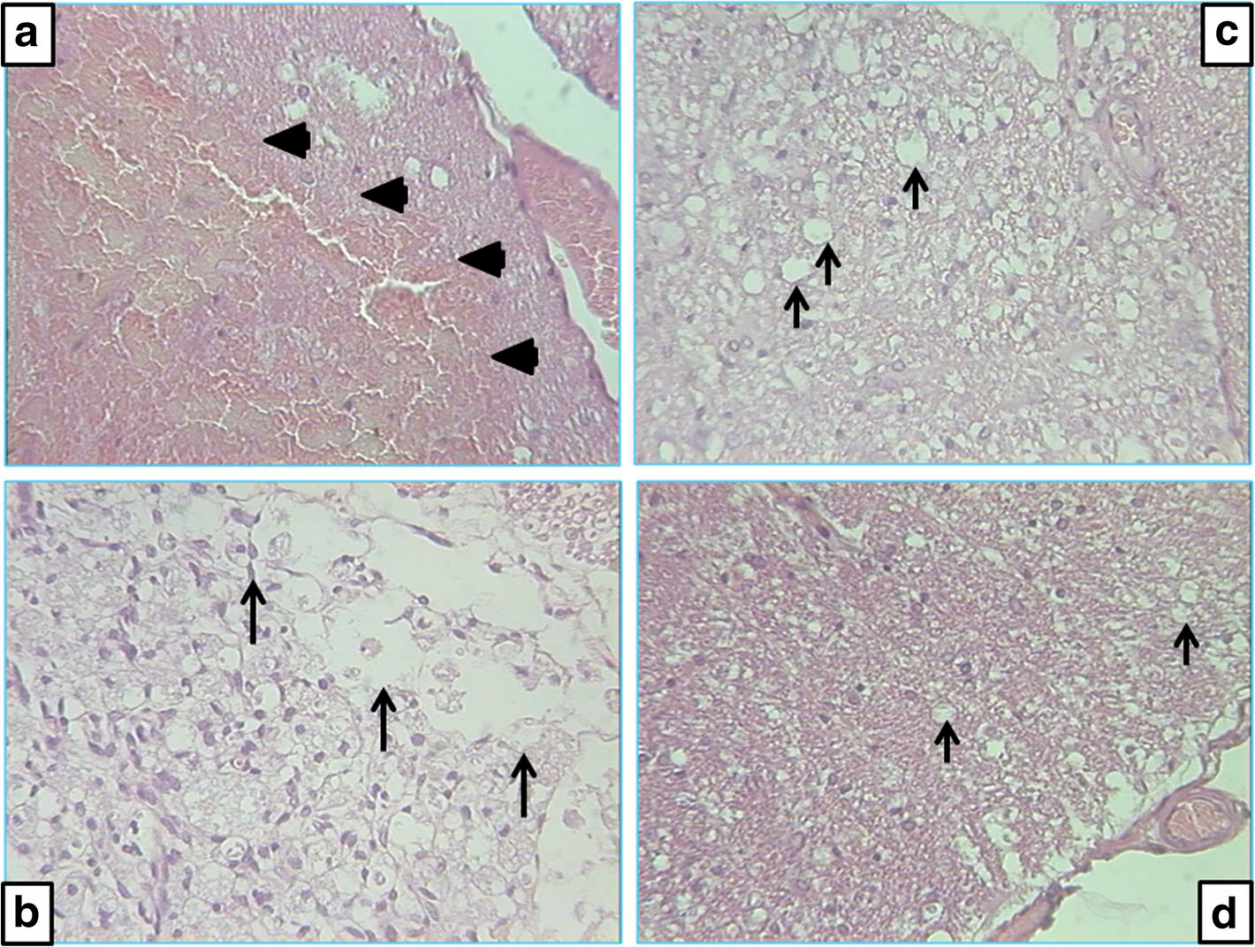
Microscopic presentation of lateral columns of rat spinal cord at the lesion site. **a** Ten minutes after injury, all animals have numerous fields of hemorrhage—arrowheads. **b**–**d** Histology 30 days after injury: **b** control animals, numerous confluent vacuoles—arrows; **c** BPC 157 2 μg/kg, few small vacuoles—arrows; **d** BPC 157200 μg/kg, only occasionally small vacuoles—arrows. Staining H&E, magnification × 300

**Fig. 5:**
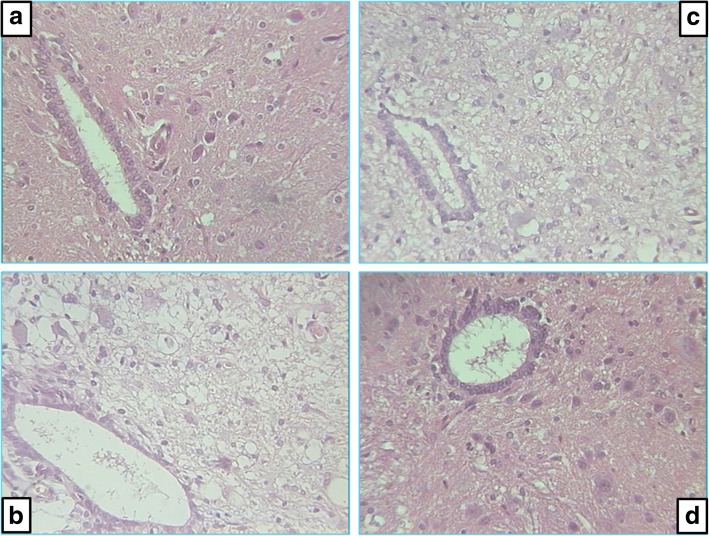
Microscopic presentation of posttraumatic spinal cord changes in the intermediate gray matter at the lesion site. **a** Ten min after injury, all animals have no difference compared to healthy animals. **b**–**d** Histology 30 days after injury: **b** control animals, huge edema and loss of neurons; **c** BPC 157 2 μg/kg, minimal edema changes and occasionally loss of neurons; **d** BPC 157 200 μg/kg, no difference to healthy animal. Staining H&E, magnification × 300

Before the initiation of therapy, at 10 min after injury induction, a large hemorrhagic zone was present over the lateral and posterior white columns in all of the rats, but there were no changes in the gray matter. Notably, after the application of saline or BPC 157, the injury progression in the rats from the different experimental groups was fundamentally different. Beginning on day 7, vacuoles and the loss of posterior and lateral spinal column tracts were observed instead of hemorrhagic areas in all controls, disturbances that were largely counteracted in the BPC 157-treated rats (Table 1 and Fig. 4). Likewise, beginning on day 7, the controls exhibited edema and the loss of neurons in the anterior horn and intermediate gray matter, disturbances that were largely counteracted the in BPC 157-treated rats (Table 2 and Fig. 5).

**Table 1:** Histology of lateral and posterior columns of spinal cord in 7 different time points, from 10 min till 360 days, after spinal cord injury

Treatment group		Time after spinal cord injury (min/med/max)						
		10 min.	7 days	15 days	30 days	90 days	180 days	360 days
Control saline 5 ml/kg b.w. i.p.	Hemorrhage	2/2.5/3	0/0/0	0/0/0	0/0/0	0/0/0	0/0/0	0/0/0
	Vacuoles	0/0/0	2/2.5/3	1/3/3	2/2/2	2/2/2	2/2.5/3	2/2.5/3
	Necrosis	0/0/0	1/2.5/3	2/3/3	2/3/3	2/3/3	1/3/3	1/3/3
Pentadecapeptide BPC 157 200 μg/kg b.w. i.p.	Hemorrhage	2/2.5/3	0/0/0	0/0/0	0/0/0	0/0/0	0/0/0	0/0/0
	Vacuoles	0/0/0	1/1/1*	1/1/1*	0/1/2*	1/1/1*	1/1/2*	1/2/2*
	Necrosis	0/0/0	0/2/2	0/0/2*	1/1/2*	0/0/1*	1/1/1*	0/0/1
Pentadecapeptide BPC 157 2 μg/kg b.w. i.p.	Hemorrhage	2/2.5/3	0/0/0	0/0/0	0/0/0	0/0/0	0/0/0	0/0/0
	Vacuoles	0/0/0	1/1/1*	1/1/1*	0/1/2*	1/1/1*	1/1/2*	1/2/2*
	Necrosis	0/0/0	0/2/2	0/0/2*	1/1/2*	0/0/1*	1/1/1*	0/0/1
Laminectomy	Hemorrhage	–	–	–	–	–	–	0/0/0
	Vacuoles	–	–	–	–	–	–	0/0/0
	Necrosis	–	–	–	–	–	–	0/0/0

Semiquantitatively evaluated intensity of (a) hemorrhagic zone, (b) vacuoles, and (c) loss of tracts in lateral and posterior columns of spinal cord (0—no changes; 1—small or focal changes; 2—moderate changes; 3—numerous confluent changes)

*Statistically significant difference BPC 157 groups vs control group, *P* < 0.025

**Table 2:** Histology of anterior horn and intermediate gray matter of spinal cord in 7 different time points, from 10 min till 360 days, after spinal cord injury

Treatment group		Time after spinal cord injury (min/med/max)						
		10 min.	7 days	15 days	30 days	90 days	180 days	360 days
Control saline 5 ml/kg b.w. i.p.	Hemorrhage	0/0/0	0/0/0	0/0/0	0/0/0	0/0/0	0/0/0	0/0/0
	Edema	0/0/0	1/1.5/2	1/3/3	3/3/3	3/3/3	3/3/3	3/3/3
	Necrosis	0/0/0	1/1.5/2	1/3/3	3/3/3	3/3/3	3/3/3	3/3/3
Pentadecapeptide BPC 157 200 μg/kg b.w. i.p.	Hemorrhage	0/0/0	0/0/0	0/0/0	0/0/0	0/0/0	0/0/0	0/0/0
	Edema	0/0/0	0/1/1	0/1/1*	0/1/3*	1/1/1*	1/1/1*	2/2/2*
	Necrosis	0/0/0	0/1/1	1/1/1	0/1/3*	0/0/2*	3/3/3	3/3/3
Pentadecapeptide BPC 157 2 μg/kg b.w. i.p.	Hemorrhage	0/0/0	0/0/0	0/0/0	0/0/0	0/0/0	0/0/0	0/0/0
	Edema	0/0/0	0/1/1	0/0/2*	1/2/2*	1/1/1*	1/1/1*	2/2/2*
	Necrosis	0/0/0	1/1/1	0/0/3	1/2/2*	0/0/2*	3/3/3	3/3/3
Laminectomy	Hemorrhage	–	–	–	–	–	–	0/0/0
	Edema	–	–	–	–	–	–	0/0/0
	Necrosis	–	–	–	–	–	–	0/0/0

Semiquantitatively evaluated intensity of (a) hemorrhagic zone, (b) edema, and (c) loss of neurons in anterior horn and intermediate gray matter of spinal cord (0—no changes; 1—small or focal changes; 2—moderate changes; 3—numerous confluent changes)

*Statistically significant difference between both pentadecapeptide BPC 157 groups and control group, *P* < 0.025

**Fig. 6:**
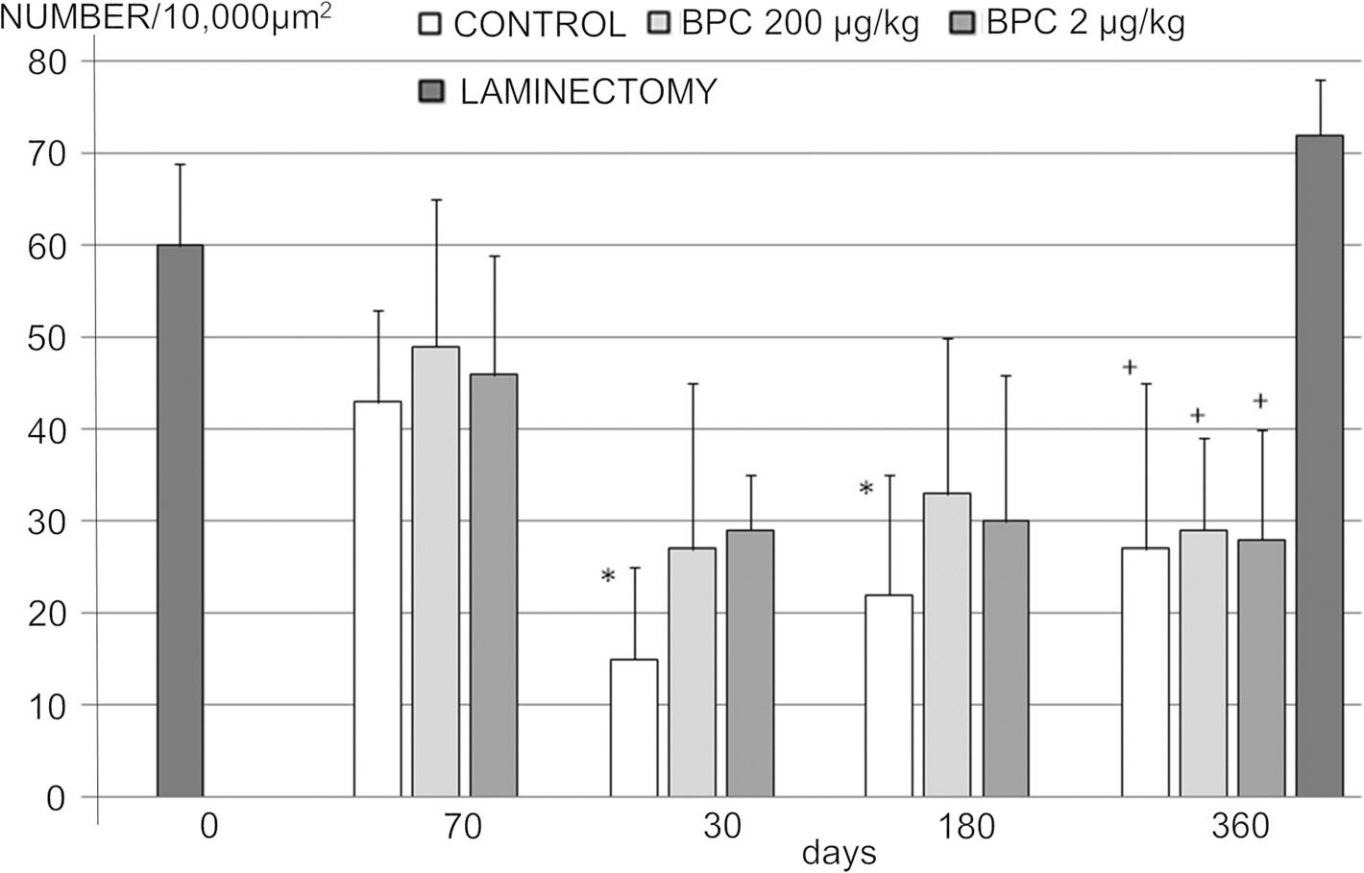
A number of myelinated axons with diameter ≥ 7 μm in rat caudal nerve per 10,000 μm^2^ in corresponding days. Columns present mean and vertical bars standard deviation. *P* value *P < 0.05 BPC 157 groups vs. saline. ^**+**^*P* < 0.05 saline and BPC 157 groups vs. laminectomy animals

While the significance of this finding remains to be determined, it is probably worth mentioning that a decrease in the number of large myelinated axons in rat caudal nerves was observed in all animals until day 30, with a markedly greater number in controls and fewer in injured rats that received BPC 157 treatment. Interestingly, after 180 days, recovery occurred, and the number of large myelinated axons in the controls reached that in the BPC 157-treated rats, and this finding persisted through the end of the experiment (Fig. 6).

### Electrophysiology results

Based on a well-known phenomenon in peripheral nerve injury (i.e., as the number of preserved motoneurons decreases, the MUP (giant potential) in the tail muscle increases), it is conceivable that the BPC 157-treated rats that underwent spinal cord injury and were subjected to EMG recordings exhibited a markedly lower MUP in the tail muscle than that in the corresponding controls (Table 3). Consistently, the motor nerve conduction study confirmed the absence of demyelinated processes in the tail caudal nerves after spinal cord injury (the CMAP showed normal biphasic potentials, similar amplitudes, and similar conduction velocities in all of the rats) (Table 4).

**Table 3:** Voluntary electromyography (EMG) of rat segmental tail muscle, in 4 different time points, from 7 days till 360 days, after spinal cord injury

Group		Mean ± SD				
			Time of EMG recording after spinal cord injury			
			7 days	30 days	180 days	360 days
Healthy control	Amp./mV	100 ± 40				
Saline 5 ml/kg b.w. i.p.	Amp./mV		180 ± 55	370 ± 52	575 ± 300	460 ± 175^+^
BPC 157 200 μg/kg b.w. i.p.	Amp./mV		200 ± 50	200 ± 80*	290 ± 150*	300 ± 65*^+^
BPC 157 2 μg/kg b.w. i.p.	Amp./mV		210 ± 25	170 ± 25*	270 ± 110*	280 ± 70*^+^
Laminectomy	Amp./mV					125 ± 30

Voluntary electromyography of rat segmental tail muscle. *Amp*. amplitude of motor unit potential (MUP) in millivolts (mV), *Mean* arithmetic mean, *SD* standard deviation

*P value < 0.05 BPC 157 groups vs. saline

^+^*P* value < 0.05 saline and BPC 157 groups vs. laminectomy animals

**Table 4:** Stimulated electromyography (EMG) of rat segmental tail muscle, in 4 different time points, from 7 days till 360 days, after spinal cord injury

Group		Mean ± SD				
			Time of EMG recording after spinal cord injury			
			30 days	90 days	180 days	360 days
Healthy control	Amp./mV NCV/m/s	3.0 ± 0.8 37 ± 6				
Saline 5 ml/kg b.w. i.p.	Amp./mV NCV/m/s		3.8 ± 1.2 38 ± 14	3.0 ± 1.0 36 ± 12	3.0 ± 1.8 35 ± 11	2.9 ± 1.2 36 ± 10
BPC 157200 μg/kg b.w. i.p.	Amp./mV NCV/m/s		3.3 ± 1.3 33 ± 4	4.3 ± 3.0 36 ± 6	2.8 ± 2.2 31 ± 10	2.6 ± 1.5 31 ± 9
BPC 157 2 μg/kg b.w.. i.p.	Amp./mV NCV/m/s		2.9 ± 0.9 37 ± 8	1.9 ± 0.8 37 ± 7	3.2 ± 2.1 30 ± 9	2.4 ± 1.0 31 ± 8
Laminectomy	Amp./mV NCV/m/s					3.3 ± 1.3 41 ± 11

Stimulated electromyography of rat segmental tail muscle. Compound muscle action potential amplitude (amp.) and nerve conduction velocity (NCV). *Mean* arithmetic mean, *SD* standard deviation. No statistical difference using one-way ANOVA analysis

## Discussion

This study attempted to demonstrate that the application of the stable gastric pentadecapeptide BPC 157 (by either of the used regimens) can improve the symptoms of spinal cord injury and lead to functional recovery in rats. In general, the one-time intraperitoneal application of the stable gastric pentadecapeptide BPC 157 is much like the engraftment of neural stem cells [16] or bone marrow stromal cells [17] into the lesion site. One should consider the primary phase lesion and hemorrhaging that results from mechanical damage during SCI as well as the secondary phase lesion that lasts several hours or even several months and is accompanied by edema, hemorrhage, inflammation, and cytotoxic edema [44–47] and may extend to the white matter area and lead to white matter degeneration and damage [48, 49]. This substantiates the evidence that the spared white matter holds the key to the functional motor recovery of the hind limbs after SCI and is closely correlated with the functional restoration of the paralyzed hind limbs [50–52]. On the other hand, spontaneous and often substantial functional improvements [53–55] after partial lesioning of the spinal cord are associated with the spontaneous sprouting of axons in the corticospinal tract [56–58] and the formation of neural circuits by spared spinal cord tissue [26]; these processes lead to partial functional recovery [59] or the formation of the neural fiber connection between the central pattern generator (CPG) and interneurons in the spinal cord, which can enable rhythmic movement [60–62].

Thus, to illustrate these combining points (i.e., [13, 44, 63]), considering that white matter injury is the major cause of functional loss after SCI [45, 52], it is important to note that cysts and the loss of axons instead of hemorrhagic areas were observed in the white matter in all of the controls beginning on day 7 and that the rats exhibited a tail motor score that persisted with only small improvements, sustained debilitation, sustained tail spasticity until the end of the experiment (day 360), a decrease in the number of large myelinated axons in the caudal nerve, a higher MUP (giant potential) in the tail muscle, and a group of atrophic fibers that likely represented a large unit that acquired many fibers through collateral reinnervation and then degenerated. Autotomy that occurs long after injury may appear as pain that occurs below the level of the injury (below-level pain) [64, 65], and the late spontaneous worsening may be the result of complete deafferentation of one or several spinal segments the stimulation of the nerve plexus, or dorsal root injury [66]. Together, these findings illustrate definitive spinal cord injury with very small spontaneous improvements in functional loss.

In contrast, it is possible that the administration of BPC 157 counteracts these disturbances to lead to considerable functional recovery. The vacuoles and the loss of axons in the white matter were largely counteracted in BPC 157-treated rats (Table 1 and Fig. 3). This result suggests that BPC 157-treated rats exhibit continual improvement in motor function even before tissue recovery, as observed by microscopy assessment. The resolution of spasticity by day 15 (Fig. 2) suggests that BPC 157 administration prevents the chain of events after spinal cord injury that is mediated by the loss of local segmental inhibition and/or by an increased sensory afferent drive that results in the exacerbation of α-motoneuron activity [66]. These findings substantiate the number of large myelinated axons in the caudal nerve and the lower MUP in the tail muscle.

Likewise, autotomy was completely prevented, much like in a previous study that showed recovery in BPC 157-treated rats that underwent traumatic nerve injury [41]; this suggests the counteraction of the chain of events that otherwise leads to painful sensations and refers to denervated regions and the preservation of one or more spinal segments [41].

It is possible that BPC 157 may affect voltage-gated sodium channels (VGSCs), which play a major role in the generation and propagation of action potentials in primary afferents [67].

The abnormal processing of sensory inputs in the CNS [68]. Moreover, evidence that the compromised white matter integrity of specific spinal pathways has been linked to clinical disability [69–71], and cortical reorganization [72] should be considered in relation to the pleiotropic beneficial effect of BPC 157 administration observed in distinctive brain areas and lesions [32–40]. These beneficial effects include the counteractions of traumatic brain injury and severe encephalopathies after NSAID overdose, insulin overdose, magnesium overdose, and exposure to the neurotoxin cuprizone in a rat model of multiple sclerosis [33–41]. These beneficial effects may be due to the formation of detour circuits—which encompass spared tissue surrounding the lesion—and could reconnect locomotor circuits [69], thus enabling afferent inputs to be processed and conveyed to the cortex [73] and improving spinal reflexes, even below the injury [74].

Much like in the rats that underwent spinal cord injury recovery, rats with other disorders that are treated with BPC 157 maintain functional abilities that are otherwise impaired; for example, consciousness is maintained after brain trauma, and BPC 157 counteracts seizures, catalepsy akinesia, and severe muscle weakness [33–41, 75, 76]. The effect of BPC 157 on muscle function is combined with the counteraction of increased levels of pro-inflammatory and pro-cachectic cytokines and of downstream pathways to abolish muscle cachexia [2]. Likewise, BPC 157 ameliorates healing and recovers the impaired function of severely injured muscles that otherwise fail to spontaneously heal and plays a role after complete transection, crush, and denervation injuries [77–80] and after succinylcholine intramuscular application, muscle lesion, neuromuscular junction failure, fasciculations, paralysis, and hyperalgesia [81]. Likewise, given that the gray matter is particularly vulnerable during the primary phase [44, 63], we should note that, from day 7, the controls presented with edema and the loss of motoneurons in the gray matter, disturbances that were largely counteracted in BPC 157-treated rats (Table 2 and Fig. 4).

In summary, this effect may be the cause or a consequence of the beneficial effects of BPC 157 on related disturbances [1–11]. As demonstrated, BPC 157 counteracts free radical formation and free radical-induced lesions [32, 82–84]. An interesting point would be the use of the same dose range in BPC 157 studies [1–11]. Finally, further studies should clarify the molecular pathways involved and extend the one-time application (much like the engraftment of neural stem cells [16] or bone marrow stromal cells [17] into the lesion site) to the continuous application for the recovery of pre-existing spinal cord injury.

In conclusion, this manuscript tried to prove the therapeutic effects of BPC 157 in spinal cord injury using a rat model. Spinal cord injury recovery was achieved in BPC 157-treated rats, meaning that this therapy affects the acute, subacute, subchronic, and chronic stages of the secondary injury phase. Thus, despite the limitations of rat studies, the results showed that treatment with BPC 157 led to the recovery of tail function and the resolution of spasticity and improved the neurologic recovery; thus, BPC 157 may represent a potential therapy for spinal cord injury.

## Data Availability

All data are included in the manuscript.

## Abbreviations

CMAP: Compound motor action potential
CNS: Central nervous system
ECM: Cone-extracellular matrix
egr-1 gene: Early growth response-1 gene
EMG: Electromyography
FAK: Focal adhesion kinase
GLP: Good laboratory practice
HNE: 4-Hydroxynonenal
HPLC: High-pressure liquid chromatography
JAK-2: Janus kinase 2
LD1: Lethal dose 1
LTB4: Leukotriene B4
MPO: Myeloperoxidase
MPTP: 1-Methyl-4-phenyl-1,2,3,6-tetrahydropyridine
MUP: Motor unit potential
naB2: Nerve growth factor 1-A binding protein-2
NSAIDs: Non-steroid anti-inflammatory drugs
SCI: Spinal cord injury
TXB2: Thromboxane B2
